# Does nonlocal women’s attendance at antenatal clinics distort HIV prevalence surveillance estimates in pregnant women in Zimbabwe?

**DOI:** 10.1097/QAD.0000000000001337

**Published:** 2017-04

**Authors:** Katherine C. Wilson, Mutsa Mhangara, Janet Dzangare, Jeffrey W. Eaton, Timothy B. Hallett, Owen Mugurungi, Simon Gregson

**Affiliations:** a Department of Infectious Disease Epidemiology, Imperial College London School of Public Health, London, W2 1PG, UK; b AIDS and TB Department, Zimbabwe Ministry of Health and Child Care, Harare, Zimbabwe; c Biomedical Research and Training Institute, Harare, Zimbabwe

**Keywords:** antenatal clinic bias, HIV surveillance, participation bias, prevalence, Zimbabwe

## Abstract

**Objective:**

The objective was to assess whether HIV prevalence measured among women attending antenatal clinics (ANCs) are representative of prevalence in the local area, or whether estimates may be biased by some women’s choice to attend ANCs away from their residential location. We tested the hypothesis that HIV prevalence in towns and periurban areas is underestimated in ANC sentinel surveillance data in Zimbabwe.

**Methods:**

National unlinked anonymous HIV surveillance was conducted at 19 ANCs in Zimbabwe in 2000, 2001, 2002, 2004, 2006, 2009, and 2012. This data was used to compare HIV prevalence and nonlocal attendance levels at ANCs at city, town, periurban, and rural clinics in aggregate and also for individual ANCs.

**Results:**

In 2000, HIV prevalence at town ANCs (36.6%, 95% CI 34.4–38.9%) slightly underestimated prevalence among urban women attending these clinics (40.7%, 95% CI 37.6–43.9%). However, there was no distortion in HIV prevalence at either the aggregate clinic location or at individual clinics in more recent surveillance rounds. HIV prevalence was consistently higher in towns and periurban areas than in rural areas. Nonlocal attendance was high at town (26–39%) and periurban (53–95%) ANCs but low at city clinics (<10%). However, rural women attending ANCs in towns and periurban areas had higher HIV prevalence than rural women attending rural clinics, and were younger, more likely to be single, and less likely to be housewives.

**Conclusions:**

In Zimbabwe, HIV prevalence among ANC attendees provides reliable estimates of HIV prevalence in pregnant women in the local area.

## Introduction

Since the beginning of the 1990s, HIV prevalence estimates used to monitor the progression of the epidemic in countries with generalized epidemics have been estimated from antenatal clinic sentinel surveillance data (ANC SS) [1–3]. The World Health Organization (WHO) now recommends that routine prevention of mother-to-child transmission (PMTCT) programme data are used to generate HIV prevalence estimates as PMTCT data cover a larger and more representative geographical area and allow women to receive their test results and be referred to treatment [4,5].

In recent years, demand has grown for local-level HIV prevalence estimates to guide more efficient targeting of resources [6,7]. PMTCT programme data could be used to produce these local estimates. However, pregnant women from rural areas often attend ANCs in urban areas – for reasons including availability of a better quality of care [8–10] – which could result in underestimates of HIV prevalence in these urban locations as prevalence generally is lower in rural areas than in urban centres [11–13]. This pattern was seen in a recent small-scale study in east Zimbabwe [14] and could also distort national HIV prevalence estimates, because the UNAIDS Spectrum software (Avenir Health, Glaston-bury, Connecticut, USA) used to generate these estimates typically is applied to model urban and rural epidemics within a country separately (Stover *et al.* in this supplement), before these are combined to produce a national estimate [15]. However, it is not known whether the finding from east Zimbabwe is generalizable to the rest of the country.

In this study, we assess whether HIV surveillance prevalence estimates in Zimbabwe are distorted by attendance at ANCs by pregnant women from nonlocal areas, and test the hypothesis that ANC-based surveillance data in towns and periurban areas understate HIV prevalence in pregnant women living in these areas because of attendance by nonlocal women from surrounding rural areas where prevalence is lower.

## Methods

We assess whether women’s attendance at nonlocal ANCs distorts HIV surveillance prevalence estimates for the area by comparing HIV prevalence among women attending the ANCs from nonlocal and local areas.

For a distortion in the HIV prevalence estimates to exist, three conditions must be met. First, a difference in HIV prevalence in urban and rural areas; second, a high level of attendance by rural women at urban ANCs; and, finally, rural women attending nonlocal clinics must have the same, or lower, HIV prevalence compared with rural women generally. Therefore, to interpret the presence or absence of any observed distortion in ANC HIV prevalence estimates, we considered the following questions:

1. Is there a difference in HIV prevalence between different areas in the national ANC SS data in Zimbabwe?
2. Is there a high level of nonlocal attendance at ANCs? Particularly attendance by women from rural areas at ANCs in urban areas.
3. Do the rural women who attend ANCs in urban areas have similar demographic and socioeconomic characteristics compared with the rural women attending rural ANCs?

### Study populations

Unlinked anonymous HIV surveillance was conducted in the same 19 ANC sites, located throughout Zimbabwe, in 2000, 2001, 2002, 2004, 2006, 2009, and 2012. In 2012, an additional 35 clinics were surveyed for the first time, with the aim of increasing the geographic representativeness of the data [16]. Pregnant women aged 15–49 years, attending for ANC check-ups for the first time during their current pregnancy were included in the survey [total number of women over all surveys, 2000–2012, in the 19 clinics (*N*) =48 497; median number of women per survey =7091].

### Definitions and variables

As part of the ANC SS questionnaire, women provide information on their residential location, which was grouped as follows: urban (town or city), periurban (farm, growth point, mine), and rural (rural, resettlement) based on the Zimbabwean Government designation. Historically in Zimbabwe, the periurban sites characterized by high labour migration (mines, growth points, border towns, and commercial farming areas) were treated as a separate category in HIV surveillance prevalence estimates because in the early stages of the epidemic, HIV prevalence in these areas was higher than in urban and rural areas [17–19]. More recently, periurban sites (also referred to as ‘other’ areas in the Zimbabwe ANC surveillance reports) have been included in the urban category [20]. For the assessment of trends in HIV prevalence between 2000 and 2012, clinics were grouped as: urbanised (city, town and periurban) and rural, and the city, town and periurban strata were also considered separately. Local women were defined as those who attended an ANC in the same area as their residential location.

### Statistical analyses

To answer the primary research question (‘Are HIV prevalence estimates from ANC SS data representative of prevalence among women attending the clinic from the local area?’), HIV prevalence estimates and 95% confidence intervals (CIs) were calculated separately by residential location for each clinic location (city, town, periurban, and rural), and for the individual clinics where a high proportion (>20%) of women attended from nonlocal areas (11/19 clinics). Where clinic locations were considered together, HIV prevalence estimates were calculated separately for each survey year (2000, 2001, 2002, 2006, 2009, and 2012); for individual clinics, the three most recent survey rounds (2006, 2009, and 2012) were pooled at each individual clinic to increase the sample size. z-score tests were used to test whether there is a statistically significant difference in the HIV prevalence among local and nonlocal attendees at the 0.05 significance level.

To answer the question (‘Is there a difference in HIV prevalence between different areas in the national ANC SS data in Zimbabwe?’), we compared HIV prevalence estimates among pregnant women attending ANC SS clinics between the years 2000 and 2012 by clinic location (city, town, periurban, and rural); 95% CIs were calculated using the logit transformation method in Stata statistical software version 13 (Stata Corporation, College Station, Texas, USA) [21].

Second, to answer the question (‘Is there a high level of nonlocal attendance at ANCs?’), we calculated the proportion of women who attended each clinic location (city, town, periurban, and rural) who lived in the same area as the clinic and in different areas.

To answer the third question (‘Do the rural women who attend ANCs in urban areas have similar demographic and socioeconomic characteristics compared with the rural women attending rural ANCs?’), the characteristics age, highest education level achieved, occupation, and marital status were compared for women attending local clinics and those attending nonlocal clinics in the national ANC SS.

## Results

### Local representativeness of antenatal clinic sentinel surveillance HIV prevalence data

In the 2000 ANC survey, HIV prevalence in town clinics (36.6%, 95% CI: 34.4–38.9%) was lower than HIV prevalence among women attending a town ANC for the first time during their current pregnancy and who are resident in a town (40.7%, 95% CI: 37.6–43.9%) (Table 1). However, no underestimation was apparent in the town clinics in subsequent survey rounds (2002–2012). Furthermore, no underestimation was apparent at clinics in periurban areas (Table 2) or in the city (Supplemental Table 1, http://links.lww.com/QAD/B15) or rural (Supplemental Table 2, http://links.lww.com/QAD/B15) areas.

In all individual clinics with a high proportion (>20%) of women attending from nonlocal areas, overall HIV prevalence was similar to that among locally resident attendees for the three most recent surveillance rounds combined (Supplemental Table 3, http://links.lww.com/QAD/B15, results presented for individual clinics in a town or periurban area). In one of the periurban clinics ‘Kadoma’, the total HIV prevalence (16.2%, 95% CI: 14.0–18.5%) underestimates prevalence among local women (20.3%, 95% CI: 16.7–24.4%). The proportion of women attending Kadoma clinic from rural areas was low (4.6% of attendees), and HIV prevalence among urban women attending Kadoma clinic (13.8%) was lower than prevalence among women from periurban areas (20.3%). (Supplemental Table 3, http://links.lww.com/QAD/B15).

### HIV prevalence by area

**Fig. 1:**
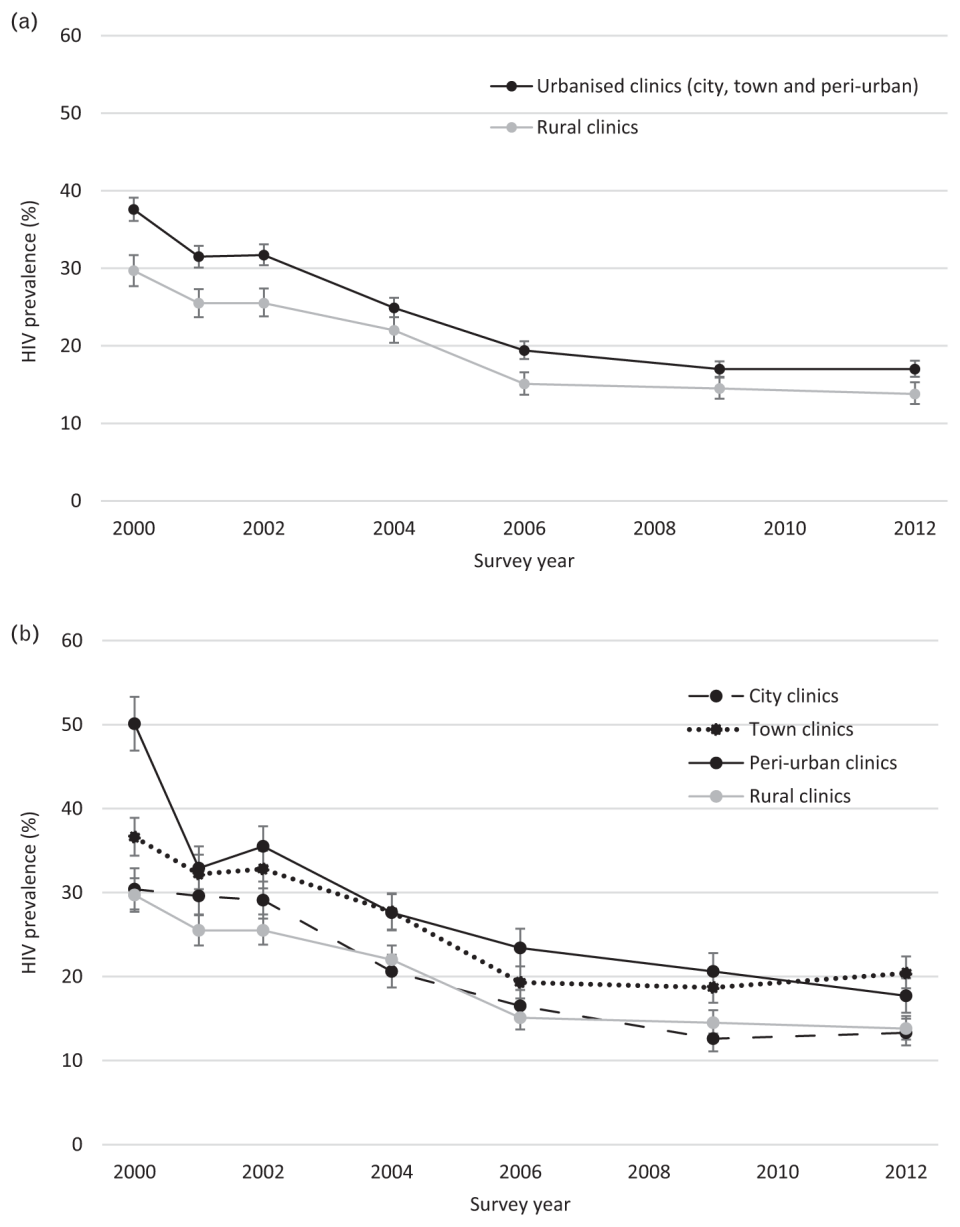
HIV prevalence among women attending antenatal clinic sentinel surveillance sites, years 2000 to 2012, by clinic location (a) urbanised (city, town and periurban) and rural; (b) city, town, periurban and rural. Error bars show 95% confidence intervals.

HIV prevalence declined overall between 2000 and 2012, and in each clinic location (city, town, periurban, and rural; Fig. 1a and b) and in each of the 19 ANC sites (data not shown). However, HIV prevalence among women attending ANCs was consistently higher in towns and periurban areas than in rural areas (Figure 1b).

The overall HIV prevalence estimate for 2012 based on the original 19 clinics included throughout the period 2000–2012 (15.7%, 95% CI: 14.8–16.5%) was similar to the estimate based on all 54 clinics included in the 2012 round of ANC SS (15.5%, 95% CI: 14.9–16.0%). However, HIV prevalence in the 35 additional clinics in 2012 compared with the original 19 clinics, was lower among urban women (14.4 vs. 16.5%, *P* =0.01) and higher among women living in periurban areas (20.2 vs. 15.8%, *P* =0.005). As was the case in the 19 clinics, HIV prevalence was higher in urban and periurban areas than in rural areas in the extended sample. (Supplemental Table 4, http://links.lww.com/QAD/B15)

### Nonlocal attendance at antenatal clinics

**Fig. 2:**
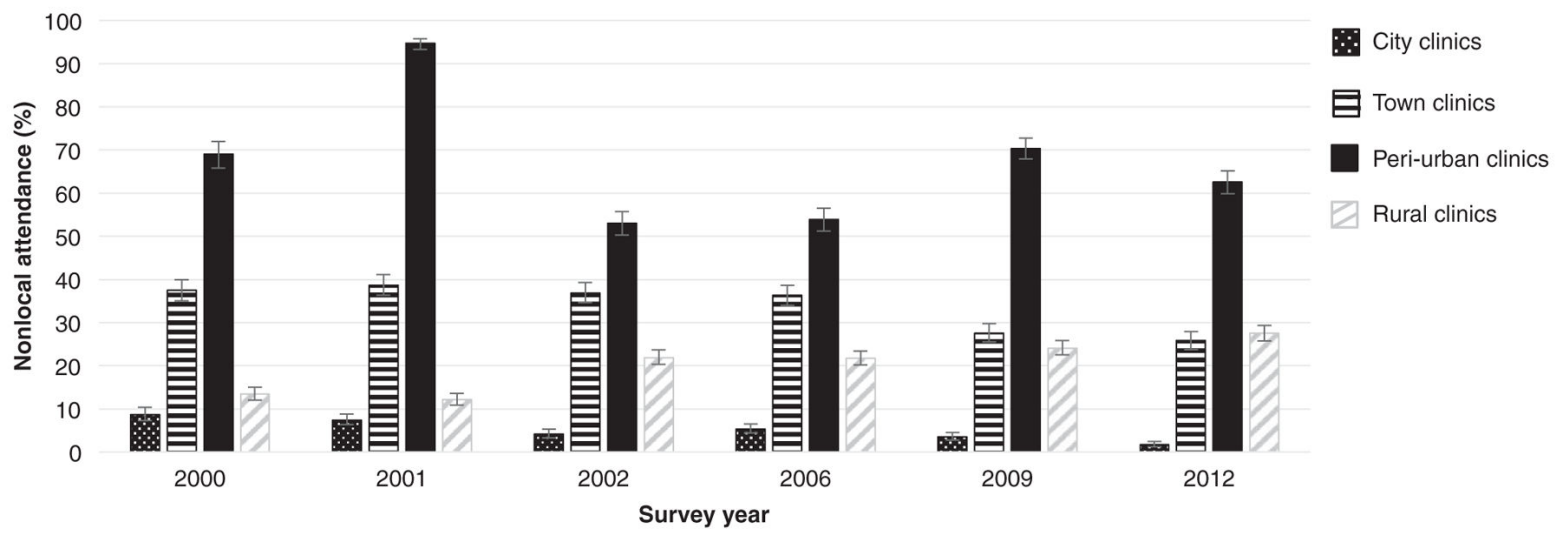
Proportion of women attending antenatal clinic sentinel surveillance sites from a nonlocal area, years 2000–2012 Error bars show 95% confidence intervals.

Just over a third (37.5%) of women attending ANCs in towns were from nonlocal areas in 2000 (Fig. 2, Supplemental Table 5, http://links.lww.com/QAD/B15). This proportion increased in 2001 (38.6%) but then reduced over time to 25.8% in 2012. Over the same period, the proportion of women attending ANCs in rural locations from nonlocal areas increased from 13.4 to 27.5%. Most of the nonlocal attendees at rural clinics came from periurban areas. Nonlocal attendance at ANCs was high in periurban areas (53–95%) but low (<10%) in cities.

### Comparison of characteristics of rural women attending local and nonlocal antenatal clinics

Even though prevalence was higher at ANC facilities in towns and periurban areas (Fig. 1b), high levels of nonlocal attendance at town and periurban ANCs by women from rural areas did not result in underestimates of HIV prevalence for pregnant women in these areas. This was because prevalence in rural women attending these ANCs was higher than among rural women attending rural ANCs (e.g. 21.8% for town ANCs vs. 14.6% for rural ANCs in 2006; Table 1 and Supplemental Table 2, http://links.lww.com/QAD/B15). This suggests that rural women who attend clinics in towns could be selected for higher risk of HIV infection. To explore this further, the results in Table 3 compare the characteristics of pregnant women from rural areas who attended town and periurban ANCs in 2012 with those who attended rural ANCs. Rural women who attended town or periurban clinics generally were slightly younger compared with rural women who attended rural clinics, and fewer were married and housewives. Among attendees at ANCs in all locations combined in 2012, the ages, education levels, occupations, and marital status of women attending their local clinics were similar to those of women attending nonlocal clinics (Table 3).

## Discussion

We hypothesized that HIV prevalence would be underestimated in towns and periurban areas where there is a high level of attendance at ANCs by pregnant women from lower prevalence rural areas. Among pregnant women attending ANC SS clinics in Zimbabwe between the years 2000 and 2012, HIV prevalence was consistently higher in towns and periurban areas than in rural areas. Also, nonlocal attendance was common at the ANCs in towns and periurban areas. Despite this, at both the aggregate clinic location and the individual clinics, we found no evidence for distortion of HIV prevalence estimates during the recent survey rounds because of women attending ANCs from nonlocal areas. The main reason for this appears to be selective attendance at ANCs in towns and periurban areas by rural women at high risk of HIV infection. We also found no evidence for distortion in HIV prevalence estimates obtained from ANCs in cities or rural areas due to attendance by women from other residential locations.

An earlier study in three districts of Manicaland province in Zimbabwe, did find evidence for underestimation of HIV prevalence in pregnant women in towns because of attendance by women from the surrounding rural areas where infection rates were lower [14]. In the Manicaland study, HIV prevalence in the rural women attending urban clinics was similar to that among those attending rural clinics and the proportion of ANC attendees at the urban clinics who lived in rural areas was greater (60–79%). One possible explanation for the discrepancy between the results of the two studies is that, in the national HIV surveillance programme, sentinel sites often include subsites which, in the case of town and periurban sites, may be located in roadside trading centres located in the surrounding rural areas but where HIV prevalence is higher than in more outlying areas.

The high prevalence of HIV infection seen among women from rural areas attending ANCs in towns and periurban areas in the national HIV surveillance sites could be because of selective attendance among rural women with high-risk sexual behaviour. This could be because these women come to urban areas to find partners and/or prefer to seek health services away from their residential area. For example, previous qualitative work in Zimbabwe has indicated that sex workers’ choice of health facility is influenced by not wanting to be recognized and stigmatized because of their trade, and therefore they may choose not to attend their nearest clinic [22]. No data were available from the national ANC SS HIV surveillance database on the sexual risk behaviour of the pregnant women. However, some of the demographic characteristics of these women (Table 3) provide indirect evidence to support this interpretation. In particular, pregnant women from rural areas who attended urban clinics were more likely to be unmarried than rural women who attended rural clinics. Single women who become pregnant may be more likely than married women to have had multiple sexual partners, and divorced, separated and widowed women typically have higher levels of sexual risk behaviour [11].

Strengths of this study include that the same 19 ANCs were included over a long time period (2000–2012), and represent areas in city, town, periurban, and rural locations. Also, in 2012, a larger number of ANCs were surveyed, which were more geographically representative; thereby, allowing us to compare estimates from this more representative sample with estimates for the 19 clinics, which had been included over a longer time period. There are also some limitations to the study. At the individual clinics, we combined the three most recent survey rounds (2006, 2009, and 2012) to increase the sample size as the uncertainty in estimates is very large when data from individual survey rounds are considered separately. However, averaging over three survey rounds could obscure effects only present in single rounds. We assumed that pregnant women attending a local clinic are a representative sample of all pregnant women living near the clinic. Although we observed similar demographic and socioeconomic characteristics between all women attending a local clinic and those attending a nonlocal clinic (Table 3), rural women who attend an ANC in a town or periurban area had a higher HIV prevalence than rural women attending a rural clinic, thus, the HIV prevalence among rural women who attend rural clinics may underestimate the HIV prevalence among all pregnant women living in rural areas. Among women aged 15–49 who had had a live birth during the 5 years preceding the 2010–11 Zimbabwe Demographic and Health Survey, 90% had received antenatal care from skilled health personnel [23]. However, it is possible that, in some areas, ANC attendance is low and HIV infection rates are higher or lower among pregnant women who do not make use of ANC services or in those who use services in different areas which could make this assumption invalid.

The findings from this study are important because they provide some reassurance that HIV prevalence estimates for pregnant women from ANC SS or from HIV surveillance based on routine PMTCT programme records are not systematically distorted because of attendance at clinics by nonlocal women. However, further work is still needed to establish whether these findings for ANC attendees can be generalized to all pregnant women and to the general population and therefore can be used to guide cost-efficient allocation of resources for HIV control in Zimbabwe. Estimates of the relative needs for resources could be distorted if there is variation by location in the overall levels and selection biases in ANC attendance, and biases in HIV prevalence among pregnant women (e.g. HIV-associated subfertility due to other sexually transmitted diseases and HIV infection [24], possibly ameliorated by antiretroviral treatment [25,26]).

In new rounds of HIV surveillance, the WHO recommends replacing ANC SS with data from routine PMTCT programmes [5]. In Zimbabwe, PMTCT services have been available in ANCs since 2002, with 95% of ANCs in 2012 offering PMTCT services. In 2011, 85% of ANC attendees accepted an HIV test as part of PMTCT services [16] and in 2012, test uptake was 95% [27]. PMTCTwill be a similar data source to ANC SS and has the potential to provide local HIV prevalence estimates for a much larger number of locations. The current findings are encouraging, therefore, in that it may be possible to use these data in resource allocation on a widespread basis. However, new biases may arise if there are high levels of refusals or HIV testing procedures are less accurate which, again, may differ in their extent between locations and will need to be considered [28].

In this study, the proportion of women from each residential location attending a particular ANC was quite variable between survey rounds, which could reflect changes in the area classification of sites or real changes in ANC usage patterns. Biases in HIV prevalence estimates in Zimbabwe could therefore occur in the future if there is an increase in the proportion of women attending an ANC from nonlocal areas where there is a difference in HIV prevalence compared with the area around the clinic. Furthermore, it is not clear whether these results are generalizable to other countries in sub-Saharan Africa with different patterns of urban–rural HIV prevalence and differences in population mobility. For example, a bias could exist in a country where HIV prevalence is much higher in urban areas than in rural areas such as Malawi. HIV prevalence from the Malawi 2010 Demographic and Health survey was 17.4% in urban areas (95% CI: 14.5–20.3%) and 8.9% in rural areas (95% CI: 8.0–9.8%) [29].

To conclude, at both the clinic location level and the individual clinic level, we found no general distortion of HIV prevalence estimates because of women attending from nonlocal areas. Therefore, ANC/PMTCT data may provide reliable estimates for HIV prevalence in pregnant women at each of these levels in Zimbabwe and could possibly be used in generating local-level HIV prevalence estimates to aid the efficient allocation of resources.

## Supplementary Material

1

## Figures and Tables

**Table 1:** HIV prevalence (HIV+ %) among women attending antenatal clinics located in towns, by residential location of the women, years 2000–2012.

Residential location	2000		2002		2006		2012	
			
	HIV+ % (95% CI)	*N*	HIV+ % (95% CI)	*N*	HIV+ % (95% CI)	*N*	HIV+ % (95% CI)	*N*
Urban	40.7 (37.6–43.9)	931	31.3 (28.5–34.1)	1040	18.5 (16.3–21.0)	1069	20.7 (18.5–23.0)	1244
Periurban	31.9 (27.0–37.3)	313	35.9 (30.9–41.3)	323	18.4 (13.9–24.0)	228	16.3 (12.3–21.4)	257
Rural	32.7 (27.1–38.8)	245	35.4 (30.1–41.2)	285	21.8 (17.9–26.2)	381	24.6 (18.7–31.5)	175
Total^a^	36.6 (34.4–38.9)	1710	32.8 (30.5–35.1)	1658	19.3 (17.4–21.2)	1682	20.4 (18.6–22.4)	1678

CI, confidence interval; N, number of women attending antenatal clinics. z-score test of HIV prevalence in local and nonlocal attendees, by survey year (P-value): 2000 (P =0.001), 2002 (P =0.06), 2006 (P =0.3), 2012 (P =0.7).

a Total includes individuals with a missing value for residential location. Number of missing (n) by survey year: 2000 (n =221; HIV+% =30.3, 95% CI: 24.6–36.7%), 2002 (n =10), 2006 (n =4), 2012 (n =2).

**Table 2:** HIV prevalence (HIV+ %) among women attending antenatal clinics located in periurban areas, by residential location of the women, years 2000–2012.

Residential location	2000		2002		2006		2012	
			
	HIV+ % (95% CI)	*N*	HIV+ % (95% CI)	*N*	HIV+ % (95% CI)	*N*	HIV+ % (95% CI)	*N*
Urban	50.1 (44.9–55.4)	343	35.8 (31.0–40.9)	360	25.1 (21.5–29.1)	498	18.4 (15.6–21.6)	647
Periurban	48.7 (42.7–54.7)	265	32.5 (28.9–36.3)	615	25.2 (22.0–28.8)	618	15.9 (12.9–19.5)	477
Rural	52.8 (46.6–59.0)	246	34.2 (29.3–39.5)	333	14.3 (10.3–19.5)	224	19.5 (13.9–26.6)	149
Total^a^	50.1 (46.9–53.3)	916	33.8 (31.3–36.4)	1310	23.4 (21.2–25.7)	1344	17.7 (15.7–19.8)	1314

CI, confidence interval; N, number of women attending antenatal clinics. z-score test of HIV prevalence in local and nonlocal attendees, by survey year (P-value): 2000 (P=0.5), 2002 (P =0.3), 2006 (P =0.1), 2012 (P =0.2).

a Total includes individuals with a missing value for residential location. Number of missing (n) by survey year: 2000 (n =62; HIV+ % =45.2, 95% CI: 33.2–57.7%), 2002 (n =2), 2006 (n =4), 2012 (n =41; HIV+ %=19.5, 95% CI: 10.0–34.7%).

**Table 3:** Demographic and socioeconomic characteristics of women who attend a local antenatal clinic and those who attend a nonlocal antenatal clinic, year 2012 (19 antenatal clinics).

	Rural women (*N* =1984)^a^		All women (*N* =7021)	
	
	Rural clinics *N* (%)	Town and periurban clinics *N* (%)	Local clinics *N* (%)	Nonlocal clinics *N* (%)
Total	1660 (83.7)	324 (16.3)	5132 (73.1)	1889 (26.9)
Age group				
15–24	801 (48.3)	167 (51.5)	2533 (49.4)	937 (49.6)
25–34	639 (38.5)	127 (39.2)	2126 (41.4)	804 (42.6)
35–49	220 (13.3)	30 (9.3)	473 (9.2)	148 (7.8)
Education				
None	14 (0.8)	6 (1.9)	27 (0.5)	9 (0.5)
Primary	560 (33.8)	93 (28.7)	1026 (20.1)	349 (18.5)
Secondary	1071 (64.6)	216 (66.7)	3948 (77.2)	1480 (78.5)
Tertiary	12 (0.7)	9 (2.8)	111 (2.2)	47 (2.5)
Occupation				
Employed	73 (4.4)	40 (12.4)	751 (14.7)	297 (15.7)
Housewife	1551 (93.6)	215 (66.6)	4027 (78.6)	1427 (75.6)
Student	1 (0.1)	1 (0.3)	54 (1.1)	10 (0.5)
Unemployed	32 (1.9)	67 (20.7)	293 (5.7)	153 (8.1)
Marital status				
Married	1622 (98.0)	285 (88.0)	4863 (95.4)	1796 (95.4)
Single	19 (1.2)	33 (10.2)	197 (3.9)	65 (3.5)
Divorced, Separated, Widowed	14 (0.8)	6 (1.9)	38 (0.7)	21 (1.1)

ANC, antenatal clinic; N, number of women attending antenatal clinics.

a Number of women (n) living in rural residential locations in 2012 who attend an ANC in the following location: city (n =16), town (n =175), periurban (n =149), rural (n =1660). Rural women attending city clinics are not included in this analysis.
