# Supplementary material for: Does nonlocal women’s attendance at antenatal clinics distort HIV prevalence surveillance estimates in pregnant women in Zimbabwe?
Source: AIDS. Author manuscript; Available in PMC 2017 Nov 9. (PMC5677598; doi:10.1097/QAD.0000000000001337)
Supplement: 1 [file NIHMS917415-supplement-1.docx]

Supplemental Table 1. HIV prevalence (HIV+ %) among women attending antenatal clinics located in cities, by residential location of the women, years 2000-2012.

|  | 2000 | |  | 2002 | |  | 2006 | |  | 2012 | |
| --- | --- | --- | --- | --- | --- | --- | --- | --- | --- | --- | --- |
| Residential  location | **HIV+ %**  **(95% CI)** | **N** |  | **HIV+ %**  **(95% CI)** | **N** |  | **HIV+ %**  **(95% CI)** | **N** |  | **HIV+ %**  **(95% CI)** | **N** |
| Urban | 30.2 (27.7-32.9) | 1201 |  | 29.2 (27.0-31.5) | 1579 |  | 16.2 (14.5-18.1) | 1618 |  | 13.3 (11.8-15.0) | 1751 |
| Peri-urban | 22.7 (9.6-45.0) | 22 |  | 41.7 (17.7-70.3) | 12 |  | 25.0 (12.9-42.9) | 32 |  | 26.7 (10.0-54.3) | 15 |
| Rural | 30.4 (21.9-40.6) | 92 |  | 22.8 (13.7-35.6) | 57 |  | 20.3 (11.9-32.6) | 59 |  | 12.5 (3.0-39.8) | 16 |
| Total^a^ | 30.4 (28.0-32.9) | 1342 |  | 29.1 (26.9-31.3) | 1652 |  | 16.5 (14.9-18.4) | 1717 |  | 13.3 (11.8-15.0) | 1795 |

^a^ Total includes individuals with a missing value for residential location. Number of missing (n) by survey year: 2000 [n=27; HIV+ %=44.4, 95% CI: 26.9-63.4], 2002 [n=4], 2006 [n=8], 2012 [n=13].

z-score test of HIV prevalence in local and non-local attendees, by survey year (P-value): 2000 (P=0.8), 2002 (P=0.6), 2006 (P=0.1), 2012 (P=0.3).

CI, confidence interval; N, number of women attending antenatal clinics.

Supplemental Table 2. HIV prevalence (HIV+ %) among women attending antenatal clinics located in rural areas, by residential location of the women, years 2000-2012.

|  | 2000 | |  | 2002 | |  | 2006 | |  | 2012 | |
| --- | --- | --- | --- | --- | --- | --- | --- | --- | --- | --- | --- |
| Residential  location | **HIV+ %**  **(95% CI)** | **N** |  | **HIV+ %**  **(95% CI)** | **N** |  | **HIV+ %**  **(95% CI)** | **N** |  | **HIV+ %**  **(95% CI)** | **N** |
| Urban | 36.9 (27.2-47.7) | 84 |  | 30.3 (21.7-40.7) | 89 |  | 12.3 (5.9-23.7) | 57 |  | 16.3 (11.1-23.4) | 141 |
| Peri-urban | 35.8 (29.3-43.0) | 187 |  | 29.6 (25.4-34.1) | 416 |  | 17.5 (14.4-21.2) | 480 |  | 15.5 (12.6-19.0) | 489 |
| Rural | 28.6 (26.5-30.7) | 1744 |  | 24.4 (22.4-26.4) | 1798 |  | 14.6 (13.1-16.3) | 1933 |  | 13.0 (11.5-14.7) | 1660 |
| Total^a^ | 29.7 (27.7-31.7) | 2050 |  | 25.5 (23.8-27.4) | 2310 |  | 15.1 (13.7-16.6) | 2479 |  | 13.8 (12.5-15.3) | 2304 |

^a^ Total includes individuals with a missing value for residential location. Number of missing (n) by survey year: 2000 [n=35; HIV+ %=34.3, 95% CI: 20.4-51.4], 2002 [n=7], 2006 [n=9], 2012 [n=14].

z-score test of HIV prevalence in local and non-local attendees, by survey year (P-value): 2000 (P=0.01), 2002 (P=0.02), 2006 (P=0.2), 2012 (P=0.09).

CI, confidence interval; N, number of women attending antenatal clinics.

Supplemental Table 3. HIV prevalence (HIV+ %) among women attending individual antenatal clinics, by residential location of the women: the years 2006, 2009 and 2012 are combined.

|  | Residential location | | | | | | | |
| --- | --- | --- | --- | --- | --- | --- | --- | --- |
|  | **Urban** | | **Peri-urban** | | **Rural** | | **Total** | |
| Antenatal clinic (location) | **HIV+%**  **(95% CI)** | **N** | **HIV+%**  **(95% CI)** | **N** | **HIV+%**  **(95% CI)** | **N** | **HIV+%**  **(95% CI)** | **N^a^** |
| Banket (town) | 21.5 (16.0-28.3) | 172 | 21.3 (18.5-24.4) | 723 | 21.6 (14.5-31.0) | 97 | 21.5 (19.0-24.1) | 1001 |
| Beitbridge (peri-urban) | 24.5 (21.6-27.7) | 758 | 29.3 (19.0-42.3) | 58 | 19.0 (13.8-25.5) | 174 | 23.8 (21.2-26.5) | 1014 |
| Bindura (town) | 16.1 (13.3-19.4) | 571 | 12.7 (9.6-16.6) | 354 | 21.3 (14.1-30.8) | 94 | 15.5 (13.4-17.8) | 1020 |
| Chiredzi (peri-urban) | 23.2 (19.5-27.3) | 457 | 19.4 (15.3-24.3) | 299 | 17.5 (13.2-22.7) | 252 | 20.8 (18.5-23.5) | 1017 |
| Gwanda (peri-urban) | 24.3 (19.8-29.5) | 304 | 23.2 (18.1-29.2) | 220 | 23.2 (19.7-27.2) | 487 | 23.5 (21.0-26.2) | 1012 |
| Kadoma (peri-urban) | 13.8 (11.2-17.0) | 542 | 20.3 (16.7-24.4) | 429 | 6.4 (2.0-18.2) | 47 | 16.2 (14.0-18.5) | 1027 |

^a^ Total includes individuals with a missing value for residential location. Number of missing (n) by antenatal clinic: Banket (n=9), Beitbridge (n=24), Bindura (n=1), Chiredzi (n=9), Gwanda (n=1), Kadoma (n=9).

z-score test of HIV prevalence in local and non-local attendees, by antenatal clinic (P-value): Banket (P=1.0), Beitbridge (P=0.3), Bindura (P=0.5), Chiredzi (P=0.5), Gwanda (P=0.9), Kadoma (P=0.003). CI, confidence interval; N, number of women attending antenatal clinics.

Supplemental Table 4. HIV prevalence (HIV+ %) among women attending antenatal clinics in 2012, by residential location of the women: comparison of 19 ANCs and additional 35 new ANCs in 2012.

|  | 19 ANCs | |  | 35 additional ANCs | |  | 54 ANCs | |
| --- | --- | --- | --- | --- | --- | --- | --- | --- |
| Residential  location | **HIV+ %**  **(95% CI)** | **N (%)** |  | **HIV+ %**  **(95% CI)** | **N (%)** |  | **HIV+ %**  **(95% CI)** | **N (%)** |
| Urban | 16.5 (15.4-17.8) | 3798 (52.9) |  | 14.4 (13.3-15.6) | 3524 (32.1) |  | 15.5 (14.7-16.4) | 7322 (40.4) |
| Peri-urban | 15.8 (13.7-18.2) | 997 (13.9) |  | 20.2 (18.3-22.2) | 1632 (14.9) |  | 18.6 (17.1-20.1) | 2629 (14.5) |
| Rural | 14.1 (12.7-15.6) | 2308 (32.2) |  | 14.4 (13.5-15.3) | 5726 (52.2) |  | 14.3 (13.6-15.1) | 8034 (44.3) |
| Missing | 18.1 (10.7-28.7) | 72 (1.0) |  | 21.2 (13.7-31.2) | 85 (0.8) |  | 19.7 (14.2-26.7) | 157 (0.9) |
| Total | 15.7 (14.8-16.5) | 7175 |  | 15.3 (14.7-16.0) | 10967 |  | 15.5 (14.9-16.0) | 18142 |

z-score test of HIV prevalence in 2012 from 19 ANCs and from the 35 additional clinics, by residential location (P-value): Urban (P=0.01), Peri-urban (P=0.005), Rural (P=0.7).

ANC, antenatal clinic; CI, confidence interval; N, number of women attending antenatal clinics.

Supplemental Table 5. Number (N) and proportion (%) of women attending antenatal clinic sentinel surveillance sites from non-local areas, years 2000-2012.

| Clinic | Residential | 2000 | 2001 | 2002 | 2006 | 2009 | 2012 |
| --- | --- | --- | --- | --- | --- | --- | --- |
| location | **location** | N (%) | N (%) | N (%) | N (%) | N (%) | N (%) |
| City | Total non-local | 114 (8.7) | 118 (7.4) | 69 (4.2) | 91 (5.3) | 63 (3.5) | 31 (1.7) |
|  | Peri-urban | 22 (1.7) | 2 (0.1) | 12 (0.7) | 32 (1.9) | 26 (1.5) | 15 (0.8) |
|  | Rural | 92 (7.0) | 116 (7.3) | 57 (3.5) | 59 (3.5) | 37 (2.1) | 16 (0.9) |
| Town | Total non-local | 558 (37.5) | 615 (38.6) | 608 (36.9) | 609 (36.3) | 474 (27.5) | 432 (25.8) |
|  | Peri-urban | 313 (21.0) | 109 (6.8) | 323 (19.6) | 228 (13.6) | 213 (12.4) | 257 (15.3) |
|  | Rural | 245 (16.5) | 506 (31.8) | 285 (17.3) | 381 (22.7) | 261 (15.1) | 175 (10.4) |
| Peri-urban | Total non-local | 589 (69.0) | 1243 (94.7) | 693 (53.0) | 722 (53.9) | 981 (70.3) | 796 (62.5) |
|  | Urban | 343 (40.2) | 346 (26.4) | 360 (27.5) | 498 (37.2) | 784 (56.2) | 647 (50.8) |
|  | Rural | 246 (28.8) | 897 (68.3) | 333 (25.5) | 224 (16.7) | 197 (14.1) | 149 (11.7) |
| Rural | Total non-local | 271 (13.4) | 279 (12.2) | 505 (21.9) | 537 (21.7) | 580 (24.1) | 630 (27.5) |
|  | Urban | 84 (4.2) | 256 (11.1) | 89 (3.9) | 57 (2.3) | 109 (4.5) | 141 (6.2) |
|  | Peri-urban | 187 (9.3) | 23 (1.0) | 416 (18.1) | 480 (19.4) | 471 (19.6) | 489 (21.4) |
